# Evidence of Alternative Splicing as a Regulatory Mechanism for Kissr2 in Pejerrey Fish

**DOI:** 10.3389/fendo.2018.00604

**Published:** 2018-10-17

**Authors:** Alejandro S. Mechaly, M. Oswaldo Tovar Bohórquez, Ariel E. Mechaly, Eda Suku, María Rita Pérez, Alejandro Giorgetti, Guillermo Ortí, Jordi Viñas, Gustavo M. Somoza

**Affiliations:** ^1^Instituto de Investigaciones Biotecnológicas-Instituto Tecnológico de Chascomús (CONICET-UNSAM), Buenos Aires, Argentina; ^2^Institut Pasteur, Platforme de Cristallographie and CNRS UMR 3528, Paris, France; ^3^Department of Biotechnology, University of Verona, Verona, Italy; ^4^Department of Biological Sciences, George Washington University, Washington, DC, United States; ^5^Laboratori d'Ictiologia Genètica, Departament de Biologia, Universitat de Girona, Girona, Spain

**Keywords:** kissr2, kissr3, kisspeptin receptors, pejerrey fish, alternative splicing, modeling, GoMoDo, fasting

## Abstract

Kisspeptin receptors are G-Protein-Coupled Receptors that regulate GnRH synthesis and release in vertebrates. Here, we report the gene structure of two kisspeptin receptors (*kissr2* and *kissr3*) in pejerrey fish. Genomic analysis exposed a gene structure with 5 exons and 4 introns for *kissr2* and 6 exons and 5 introns for *kissr3*. Two alternative variants for both genes, named *kissr2_v1* and *_v2*, and *kissr3_v1* and *v2*, were revealed by gene expression analyses of several tissues. For both receptors, these variants were originated by alternative splicing retaining intron 3 and intron 4 for *kissr2_v2* and *kissr3_v2*, respectively. In the case of *kissr2*, the intron retention introduced two stop codons leading to a putatively truncated protein whereas for *kissr3*, the intron retention produced a reading shift leading to a stop codon in exon 5. Modeling and structural analysis of Kissr2 and Kissr3 spliced variants revealed that truncation of the proteins may lead to non-functional proteins, as the structural elements missing are critical for receptor function. To understand the functional significance of splicing variants, the expression pattern for *kissr2* was characterized on fish subjected to different diets. Fasting induced an up-regulation of *kissr2_v1* in the hypothalamus, a brain region implicated in control of reproduction and food intake, with no expression of *kissr2_v2*. On the other hand, fasting did not elicit differential expression in testes and habenula. These results suggest that alternative splicing may play a role in regulating Kissr2 function in pejerrey.

## Introduction

G-protein-coupled receptors (GPCRs) play key roles in many physiological processes and have been associated with multiple human diseases (1). Since changes in normal GPCRs signaling affect many pathophysiological mechanisms, these receptors have been targets for several drug therapies (1–3). The superfamily of GPCRs is characterized by having seven-transmembrane (7TM) α-helices connected by three extracellular loops (ECLs) and three intracellular loops (ICLs), an extracellular amino-terminal segment, and an intracellular carboxy-terminal tail (4). According to their amino acid sequence, GPCRs are classified into five major classes (families): (i) A or Rhodopsin-like (the largest group), (ii) B or Secretin receptor, (iii) C or Metabotropic Glutamate receptor, (iv) adhesion, and (v) frizzled/taste (5).

In 2001, a member of the Rhodopsin family, the kisspeptin receptor KISS1R (previously named GPR54) was shown to be activated by polypeptides kisspeptin-54,-14,-13, and-10 (6–8). A few years later, kisspeptin and its receptor were regarded as essential regulators of the reproductive axis, since hypogonadotropic hypogonadism in both humans and mice was shown to be associated with mutations of *KISS1R* (9, 10). Moreover, kisspeptin and its receptor were linked to other functions such as insulin secretion (11), vasoconstriction (12), tumor biology and the metastatic process (13), antioxidant function in oxidative stress (14), anticoagulation (15), and brain sex differentiation (16).

During the last decade, many studies on vertebrate reproduction readily identified kisspeptin receptors in a large number of species facilitated by the highly conserved structure of their 7TMs domains. Only one gene, known now as *Kiss1r*, has been reported in placental mammals (17). In contrast, two paralogous *kissrs* (namely *kissr2* and *kissr3)* are frequently detected in teleost fishes, likely originating from the teleost-specific whole-genome duplication in the common ancestor of teleosts (17–19). Of particular interest is the case of the Senegalese Sole (*Solea senegalensis*), for which a single *kissr2* but no *kissr3* genes were reported (20). However, two *kissr2* transcripts were identified in this species, a short one named *kissr2_v1* corresponding to the normally-spliced messenger, and a long *kissr2_v2* (putatively non-functional) transcript characterized by retaining the entire intron 3 (20). Subsequently, the presence of alternatively spliced variants of *kissr2* was documented in other teleost species such as the Southern Bluefin Tuna (*Thunnus maccoyii*) and the Yellowtail Kingfish (*Seriola lalandi*) (21). Alternative transcripts or spliced variants for *kissr3* (previously known as *kiss1rb*) also were described for zebrafish (*Danio rerio*) (22) and the European eel (*Anguilla anguilla*) (23). Although some studies have suggested preservation of functionality of truncated GPCR transcripts (22, 24), evidence to support this claim remains elusive.

A functional relationship between food intake and reproduction has been well established among fishes (25), however little is known about the putative role, if any, that the kisspeptin system may have to modulate this interaction. The first report supporting such a role was a study on Senegalese sole that showed up-regulation of hypothalamic *kiss2* and *kissr2* expression during starvation, in concert with an increase of transcript levels of gonadotropins in the pituitary (26). Similarly, food restriction was shown to enhance hypothalamic *kiss2* and *kissr2* gene expression and to increase mRNA levels of follicle-stimulating hormone and luteinizing hormone β subunits (*fshb* and *lhb*) in the pituitary of the European sea bass (*Dicentrarchus labrax*) during spermatogenesis (27).

In the present study, we report the predicted structure of two *kissr* genes, *kissr2* and *kissr3* in pejerrey fish (*Odontesthes bonariensis*). We also identify new alternative spliced variants for each receptor and provide preliminary evidence suggesting loss of function of variants due to intron retention. We also test the expression pattern of *kissr2_v1* and *kissr2_v2* in pejerrey hypothalamus after fasting, because a similar condition was reported to increase not only hypothalamic *kiss2* but also *kissr2* in *S. senegalensis* (26).

Our findings suggest a novel *kissr2* gene regulatory mechanism in the hypothalamus involving expression of alternatively spliced variants with intron retention that produce potentially non-functional proteins.

## Materials and methods

### Fish and tissue sample collection for gene characterization

Adult pejerrey (*Odontesthes bonariensis*) were maintained in outdoor tanks of the IIB-INTECH aquatic facilities under natural photoperiod and water temperature of 17 ± 2°C. They were fed daily with fish commercial pellets (Shulet®, Argentina). For each of the experimental purposes, fish were anesthetized with an over-dose of benzocaine and then decapitated. The different tissues and organs were quickly dissected under clean conditions, immediately frozen in liquid nitrogen and then stored at −80°C until used. The fish were handled in accordance with the UFAW Handbook on the Care and Management of Laboratory Animals (http://www.ufaw.org.uk) and IIB-INTECH internal institutional regulations. These animal protocols were approved by a professional board.

### Gene structure of *kissr2* and *kissr3* in pejerrey

The BLAST 2.2.29 algorithm was used to retrieve the genomic sequences of *kissr2* and *kissr3* from the pejerrey genome database (28) using pejerrey partial sequences of both genes (29). To assess the genomic gene structure, the coding sequences of both genes (29) were amplified by the primer combinations kissr2-Ex1-F/kissr2-Ex5-R and kissr3-Ex1-F/kissr3-Ex6-R (Table 1) using complementary DNA (cDNA) as template. Genomic DNA was extracted according to Aljanabi and Martinez (30). Briefly, a portion of muscle (~50 mg of tissue) was obtained and homogenized in 400 μl of saline buffer (NaCl 0.4 M, Tris-HCl 10 mM pH 8, EDTA 2 mM pH 8), then 40 μl of SDS 10% and 8 μl of proteinase K (10 mg/ml) were added and the mixture incubated at 65°C for 1 h. After incubation, 300 μl of NaCl 6 M were added and the solution was centrifuged at 10,000 g for 15 min. Finally, the supernatant was obtained and precipitated with 95% ethanol, washed with 70% ethanol and dissolved in 50 μl of ultrapure water (Invitrogen™, USA).

**Table 1:** Gene-specific primers used to build the gene structure organization, detection of alternative splicing and qPCR studies.

**Gene**	**Primer sequence**	**Amplicon size (bp)**	**Primer name**
*kissr2*	TCATTTCCCCACAACAACCTCTTT		kissr2-Ex1-F
	TAGATTTACCCCATTATCATTATTG	1,176	kissr2-Ex5-R
	GGGACCGCTGTTATGTGAC		kissr2-Ex3-F
	GCCGTACCAGTAACCCTCCT	158/256	kissr2-Ex4-R
*kissr2_v1*	GTCAGCATCTGCATTTGGATTGGC		kissr2-Ex3-4-F
	CTCCACGCAGTATTGTCTCGGG	97	kissr2-Ex4-R1
*kissr2_v2*	CAATGACAAATCAAAACCAATCA		kissr2-Int3-F
	GCCGTACCAGTAACCCTCCT	109	kissr2-Ex4-R
*kissr3*	ATGGCTGCAGAATCAGGAG		kissr3-Ex1-F
	TTAGGACCCAGATGAAAGAA	1,107	kissr3-Ex6-R
	ACCATTATCGCCTGTTACGC		kissr3-Ex4-F
	ATTGCTGTTGCTCGCTTTG	110/189	kissr3-Ex5-R
*kiss2*	CAGAGAGAGCGACGACCAG		kiss2-F
	AGAGAAAGAGGGGCGAAAAC	161	kiss2-R
*lhb*	CATCCAGTGGAAGCAACCATCT		lh-F
	CGTGCACACACTTTGGTACATGT	96	lh-R
*fhsb*	GGCTGCCACCTCGACTGTTAT		fsh-L
	TGAAGCACAGTCCTTCACATATGG	103	fsh-R
*ef1*	AGAAATCCGTCGTGGATACG		ef1-F
	TGATGACCTGAGCGTTGAAG	83	ef1-R
*β-actin*	GCTGTCCCTGTACGCCTCTGG		bactin-F
	GCTCGGCTGTGGTGGTGAAGC	200	bactin-R
	AAGGCCAACAGGGAAAAGAT		bactin-F1
	GTCCCCATTTCTTGCTCAAA	350/≃800	bactin-R1

*Different amplicon sizes for a pair of primers represent the difference of the isoforms*.

### RNA isolation and cDNA synthesis

Total RNA was isolated with TRIZOL® Reagent (Invitrogen™, USA) from different organs and tissues. Quality of RNA was assessed in all samples using 1% agarose-formaldehyde gels and their quantity measured with a Biotek H1 synergy analyzer (Biotek®, USA). All RNAs were treated with DNaseI (Invitrogen™, USA) to remove any possible genomic DNA contamination. Subsequently, cDNA was synthesized using ~500 ng of RNA with Superscript II Reverse Transcriptase (Invitrogen™, USA), RNaseOUT recombinant ribonuclease inhibitor (Invitrogen™, USA), and oligo dT universal adaptor primer in 20 μl reaction volume, following the manufacturer's instructions.

### Detection of *kissr2* and *kissr3* alternatively spliced variants in pejerrey

Complementary DNA from hypothalamus, testis, and habenula were used as templates to search for alternatively spliced variants of *kissr2* and *kissr3* in adult animals by RT-PCR. In the case of *kissr2*, primers located between exons 3 and 4, including intron III (kissr2-Ex3-F and kissr2-Ex4-R) were specifically designed to amplify both splicing variants. For *kissr3*, primers between exons 4 and 5, including intron IV were designed with the same purpose (kissr3-Ex4-F and kissr3-Ex5-R; Table 1). PCR amplifications of these transcripts were performed using an initial heat denaturation step at 94°C for 5 min, followed by 40 cycles of 30 s at 94°C, 30 s at 55°C, and 1 min at 72°C, and finished with a final extension step at 72°C for 5 min. PCR products were visualized by 1% agarose gel electrophoresis.

The tissue expression pattern of *kissr2* and *kissr3* splicing variants were analyzed by RT–PCR in different pejerrey tissues and organs. For the brain we separated three different regions after dissection: rostral (including the olfactory bulbs, telencephalon, and preoptic area), medial (including the optic tectum, thalamus, pineal gland, and hypothalamus), and caudal (cerebellar body, vagal lobe, and the medulla oblongata). The following tissues and organs also were analyzed for specimens of both sexes: pituitary gland, gonads, olfactory epithelium, retina, lateral line, liver, gills, muscle, heart, foregut, midgut, hindgut, kidney, and spleen. Total RNA isolation and cDNA synthesis were carried out following the above described protocols. The resulting cDNA was used to amplify *kissr2* and *kissr3* by RT-PCR using GoTaq® DNA Polymerase (Promega, USA). The PCR cycling conditions were: 5 min at 94°C; 40 cycles of 30 s at 94°C, 30 s at 60°C, 30 s at 72°C and a final extension of 5 min at 72°C. RNA quality and genomic DNA contamination in cDNA were checked by amplification of the β*-actin* gene with a primer combination encompassing an intron (bactin-F1 and bactin-R1) (Table 1, Figure 3C). No template controls (NTC) were included to ensure that no contamination occurred. All PCR products were run on 1% agarose gels.

### Kissr homology model and docking

The Kissr2_v1 and Kissr3_v1 structural models and their interaction with ligand peptides were inferred using GOMoDO (http://molsim.sci.univr.it/cgi-bin/cona/begin.php). The peptides were docked in the predicted binding cavities by using the Haddock program, accessible through the GOMoDo server (31). Figures depicting 2D and 3D receptor models were produced with Topdraw (32) and Chimera programs (http://www.cgl.ucsf.edu/chimera/), respectively.

### Fasting effect on *kissr2* alternative splicing

We choose the hypothalamus as a target organ to evaluate the relationship between expression levels of *kissr2_v1* and *kissr2_v2*, because differential expression of *kissr2* has been reported during fasting in other fish species (26, 27). In addition, the mRNA expression patterns of *kissr2_v1* and *kissr2_v2* (kissr2-Ex3-4-F1/kissr2-Ex4-R1 and kissr2_Int3_F/kissr2_Ex4_R primer pairs) (Table 1; Supplementary Figure 1) were analyzed in the testes and habenula, together with *lhb* and *fshb* in the pituitary gland. Adult pejerrey males (119.86 ± 9.21 g) were transferred to 300 l indoor tanks with similar conditions to outdoor tanks. Fish were distributed into different tanks (*n* = 8 per tank) and acclimated for 1 week before starting experimental manipulations. After acclimation, one group was starved and the other (control) was feed *ad libitum* three times a day for 15 days. These experimental procedures were performed in duplicate. Fish were sacrificed as described above, the hypothalamus and habenula were dissected from the brain, and the pituitary gland and testes also were sampled as controls. Total RNA was extracted and cDNA obtained according to described protocols. The expression patterns were analyzed by relative quantitative PCR (qPCR). All primers used for qPCR designed to amplify each *kissr2* splicing variant are listed in Table 1 and their quality values in Supplementary Table 1. The qPCR amplification reaction mixture contained 2 μl of diluted cDNA (1:20), 300 nM of each primer, and 5 μl of FastStart Universal SYBR Green Master (Rox) (Roche Diagnostics, Germany) in a final volume of 10 μl. The thermal cycling conditions were 95°C for 10 min, 40 cycles at 95°C for 15 s, and 60°C for 1 min. At the end of the PCR cycles, the qualities of qPCR products were analyzed using a dissociation curve step to confirm that only a single PCR product was amplified. NTC reactions for every primer pair also were included on each reaction plate to ensure no external DNA contamination. The amplification efficiency (E) of each primer set/target gene was assessed as E = 10^(−1/slope)^ as determined by linear regression of a series of dilutions of the input RNA. The qPCR reactions were performed with a Step-one Real-time PCR System (Applied Biosystems, USA). Fold change (the relative quantification, RQ) was calculated from the ΔΔCt (33). Determinations were carried out in technical triplicates for all the genes and normalized against the reference genes (*ef1* and β*-actin)* (ef1-F/ef1-R and bactin-F/bactin-R primer pairs) (Table 1). The RQ values for each sample were averaged and the standard error of the mean (SEM) was calculated. Controls without cDNA template (Supplementary Figure 1B), and a melt curve analysis (Supplementary Figure 1C) were used to determine the specificity of the amplification.

### Statistical analyses

The SPSS v20 program was used to perform all the statistical analyses and results were expressed as mean ± SEM. Statistical significance of pairwise comparisons of body weight and mRNA levels was determined using Student *t*-test (^*^p < 0.01, ^*^p < 0.05).

## Results

### Genomic structure of *kissr2* and *kissr3* genes in pejerrey

**Figure 1:**
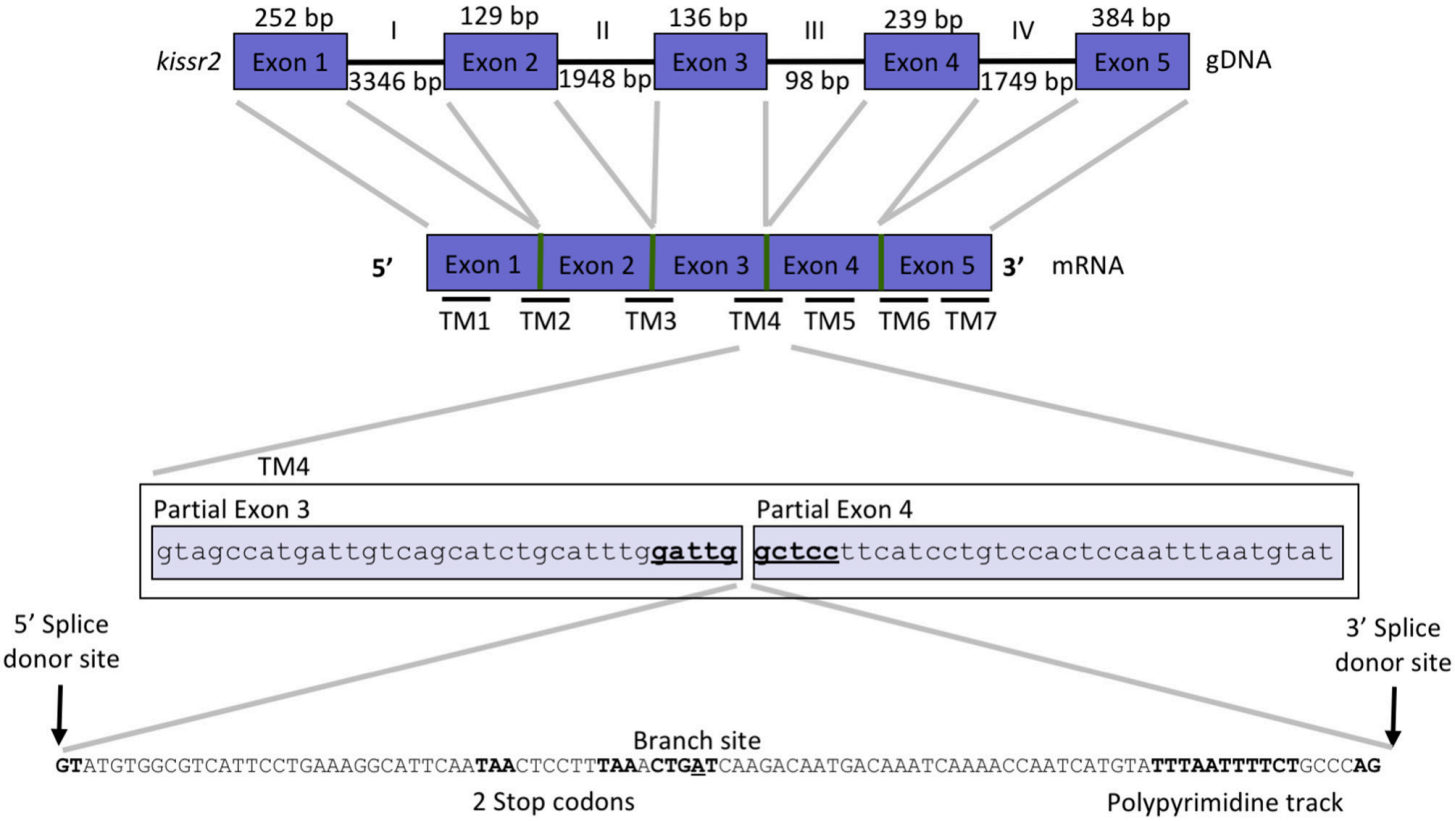
Gene structure of *kissr2* in pejerrey. Exons are displayed as blue boxes and introns as thick lines. Thinner horizontal lines at the bottom of the cDNA scheme, represent the site of the transmembrane domains (TMs). A detail of pejerrey intron 3 of *kissr2* sequence with features consistent with the presence of a mechanism for alternative splicing is shown.

**Figure 2:**
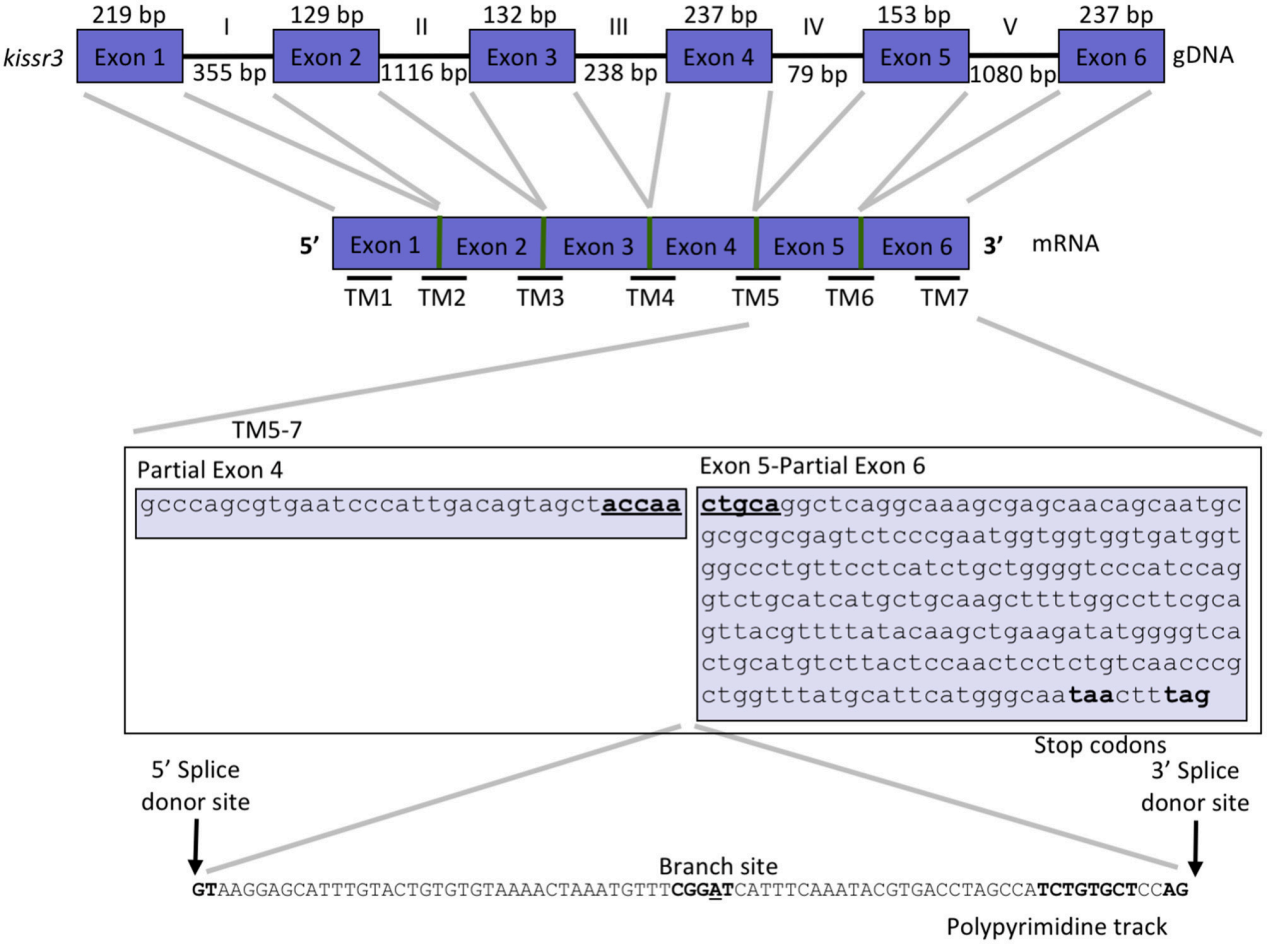
Gene structure of *kissr3* in pejerrey. Exons are displayed as blue boxes and introns as thick lines. Thinner horizontal lines at the bottom of the cDNA scheme, represent the site of the transmembrane domains (TMs). A detail of pejerrey intron 4 of *kissr3* sequence with features consistent with the presence of a mechanism for alternative splicing is shown.

We used the cDNA sequences of the coding region of *kissr2* in pejerrey, previously reported by Tovar Bohórquez et al. (29), to scan the pejerrey genome (28) and locate the genomic sequence of the duplicated *kissr* genes, *kissr2* and *kissr3*. The gene structures were constructed using the nucleotide and predicted peptide sequences of *kissr2* and *kissr3* compared with known *kissr* sequences available in GenBank. Localization of the intron-exon boundaries sites was based on alignment of the cDNA and genomic DNA for each receptor. The structure of the *kissr2* gene exhibits five exons (with 252, 129, 136, 239, and 384 bp, respectively) and four introns (3,346, 1,948, 98, and 1,749 bp, respectively) (Figure 1). The structure of *kissr3* is composed of six exons (with 219, 129, 132, 237, 153, and 237 bp, respectively) and five introns (with 355, 1,116, 238, 79, and 1,080 bp, respectively) (Figure 2).

Transcriptomic sequence analysis revealed that the longer transcript *kissr2_v2* retained the entire intron 3 and displayed the presence of several consensus features of alternative splicing mechanisms in the introns: a 5′ donor splice site, potential branch points, polypyrimidine tracks and 3′ acceptor splice sites (Figure 1). Translation of the *kissr2_v2* isoform DNA to its putative amino acid sequence revealed the presence of two premature stop codons. In the case of the longer *kissr3_v2* isoform, retention of intron 4 caused a shift in the reading frame that included two premature stop codons in exon 5 (Figure 2).

### Alternatively spliced variants of *kissr2* and *kissr3*

Two sets of specific primers that amplified regions encompassing intron 3 in *kissr2* and intron 4 in *kissr3* were used for sequence analyses (Table 1). These primers generated two different amplicons for each gene (see Supplementary Figure 1A), as follows: *kissr2_v1* (158 bp), *kissr2_v*2 (256 bp), *kissr3_v1* (110 bp), and *kissr3_v2* (189 bp).

**Figure 3:**
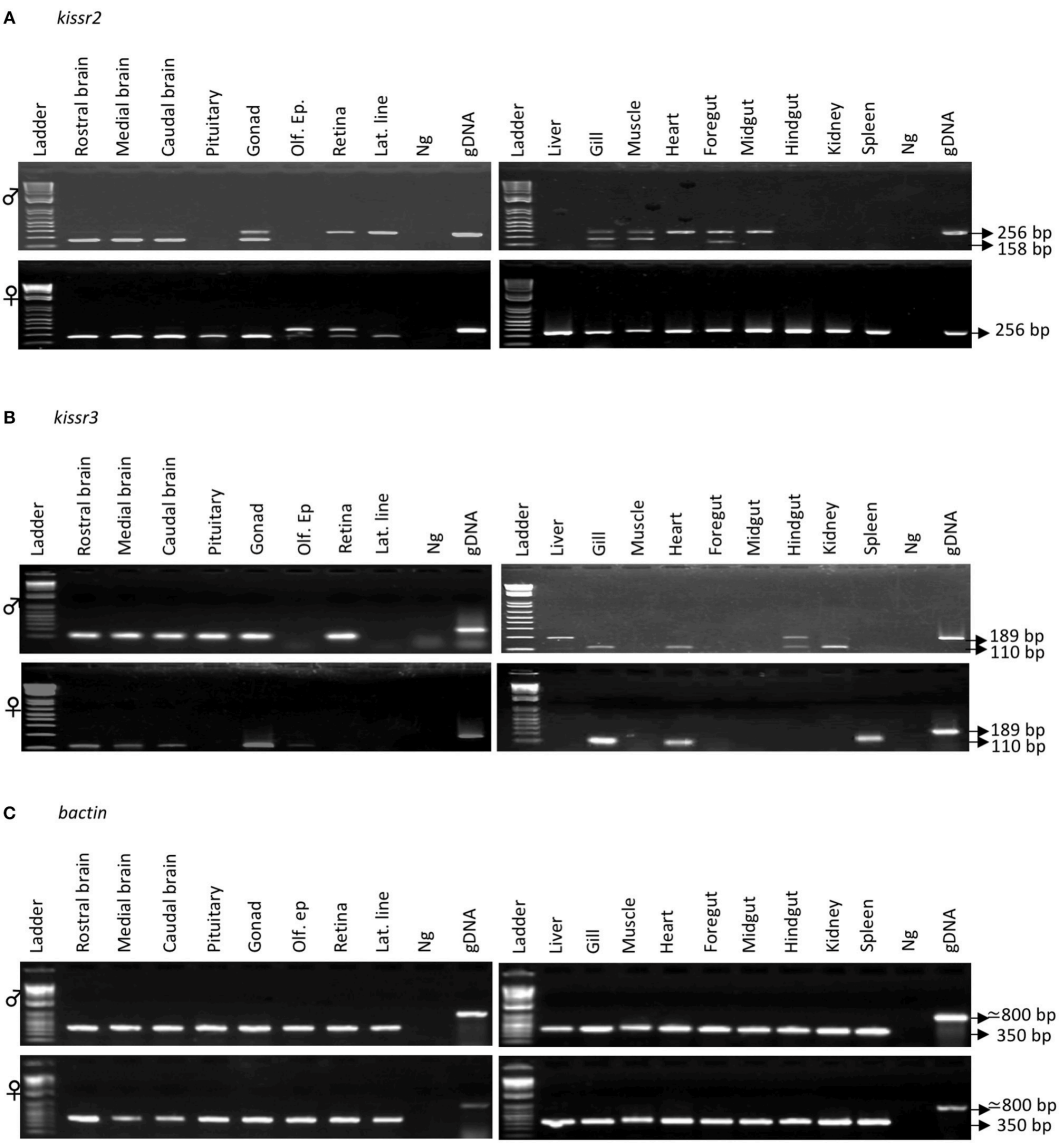
Tissue expression of the *kissr2* **(A)** and *kissr3* **(B)** was analyzed by RT-PCR in mature pejerrey. Total RNA was prepared from rostral brain, medial brain, caudal brain, pituitary, testis (in males), ovary (in females), olfactory epithelium (Olf. Ep), retina, lateral line, liver, gills, muscle, heart, foregut, midgut, hindgut, kidney, and spleen from one male and one female. Messenger RNAs of the two isoforms were detected by RT-PCR (kissr2-Ex3-F/kissr2-Ex4-R and kissr3-Ex4-F/kissr3-Ex5-R primers). Lane 1, 100 bp ladder; Lane 2-8/9, tissues cDNA; Lane 9/10, Negative control (Ng); Lane 10/11, genomic DNA (gDNA). **(C)** β*-actin* gene was amplified to check the absence of gDNA and as a control of the cDNA integrity.

In males, the rostral, medium and caudal brain, testis, gills, muscle, and foregut showed expression of both *kissr2* transcripts (_v1 and _v2). The retina, lateral line, heart, and midgut only showed *kissr2_v2* expression while pituitary, liver, hindgut, kidney, and spleen did not show expression of either spliced variant (Figure 3A). In females, *kissr2_v1* was clearly visualized in rostral, medial and caudal brain, pituitary, and gonads; and dimly in retina and lateral line, whereas *kissr2_v2* transcript was detected in the olfactory epithelium, retina, liver, gill, muscle, heart, foregut, midgut, hindgut, kidney, and spleen (Figure 3A). In the case of *kissr3*, in males, *kissr3_v1* was detected in the rostral brain, medial brain, caudal brain, pituitary, testis, retina, gill, heart, hindgut, and kidney, meanwhile *kissr3_v2* was only observed in the liver and hindgut (Figure 3B). In females, *kissr3_v1* was observed in the rostral brain, medial brain, caudal brain, ovary, olfactory epithelium, gill, heart, and spleen, while *kissr3_v2* was not detected (Figure 3B).

### Homology modeling and molecular docking of kisspeptins and their receptors

**Figure 4:**
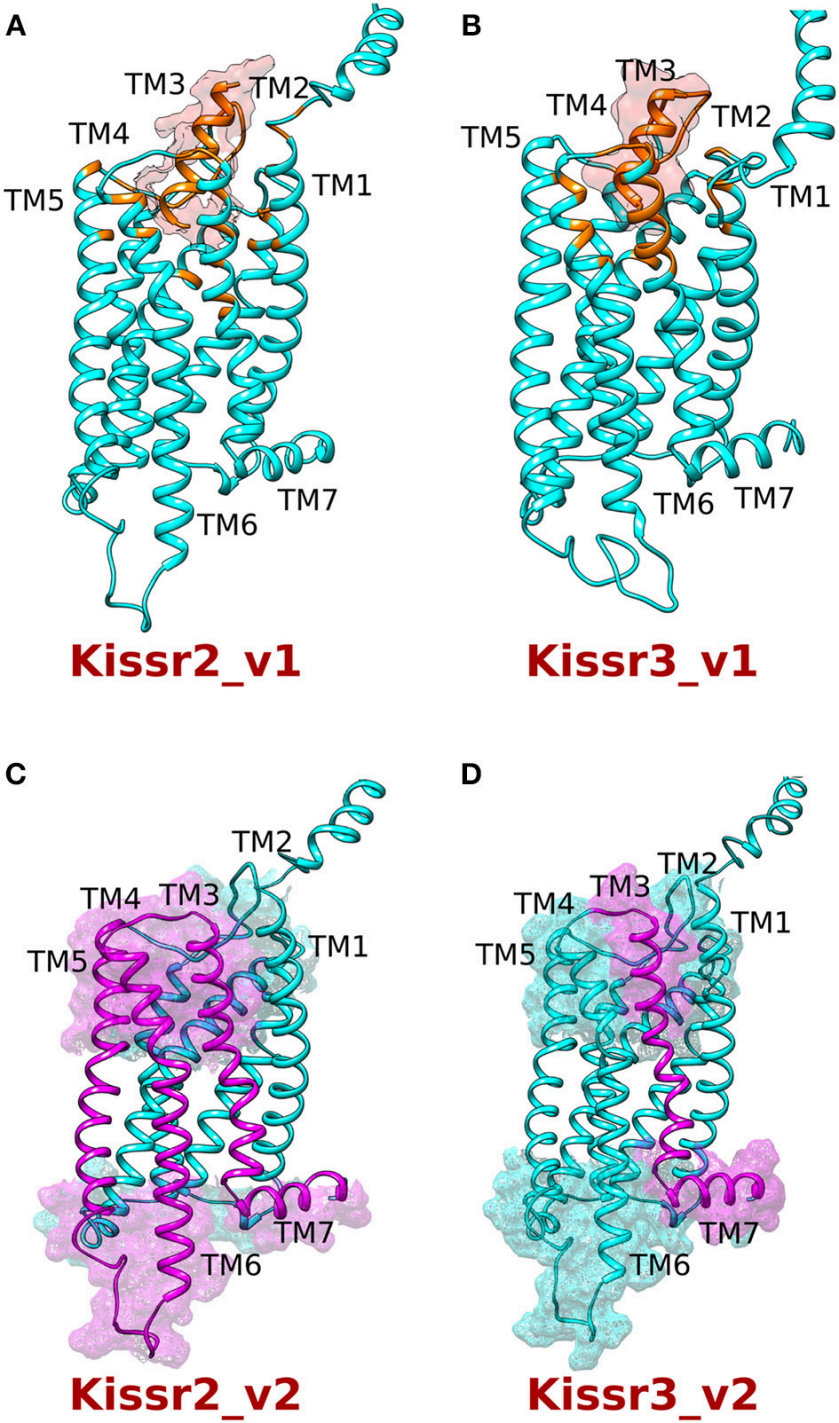
Homology modeling of the kisspeptin receptors (Kissr2 and Kissr3) with their respective peptides kiss1 and kiss2. **(A)** Kissr2_v1 receptor (cyan) and **(B)** Kissr3_v1 receptor (cyan) with their respective peptides (orange). The residues located within 5 Å from the peptides are shown in orange. **(C,D)** The truncated region of both the receptors is shown in violet. While receptor Kissr2_v2, lacks TM5-TM7 helixes; Kissr3_v2 loses TM7 and portion of the extracellular loop 3.

Homology 3D models of pejerrey Kissr2 and Kissr3-structures were built using the on-line platform GOMoDo (Figure 4, Supplementary Figure 2 for Kissr2 and Supplementary Figure 3 for Kissr3). Their respective peptides (Supplementary Figure 4) were then docked in the predicted binding cavities by using the Haddock program accessible also through the GOMoDo server (Supplementary Table 2). From the models it can be observed that: (i) in Kissr2_v1 the putative ligand binding cavity is formed by residues of TM3 (Gln125, Gln126, Val129, Gln130), ECL3 (Tyr197, Cys198, Glu200), TM5 (Gln215, Tyr220), TM6 (Leu276, Trp281, Ile284, Gln285), and TM7 (Asn311, Tyr315) (Figure 4A); and (ii) in pejerrey the Kissr3_v1 putative binding cavity is formed by residues of TM3 (Gln114, Gln115, Ala118, Gln119), ECL3 (Gln183, Thr184, Cys186), TM5 (Ser203, Tyr208), TM6 (Leu264, Trp269, Ile272, Gln273), and TM7 (His296, Tyr300) (Figure 4B). It is important to note that in both receptors the residues that are putatively crucial for ligand and G-protein binding (according to the prediction of the method used) belong to helices TM5-7, just like several other GPCRs analyzed before (34). This evidence suggests that loss of these helices in variants *kissr2_v2* and *kissr3_v2* could compromise receptor structure, function, or dimerization (Figures 4C,D).

### Effect of fasting on *kissr2_v1* and *kissr2_v2* expression levels

**Figure 5:**
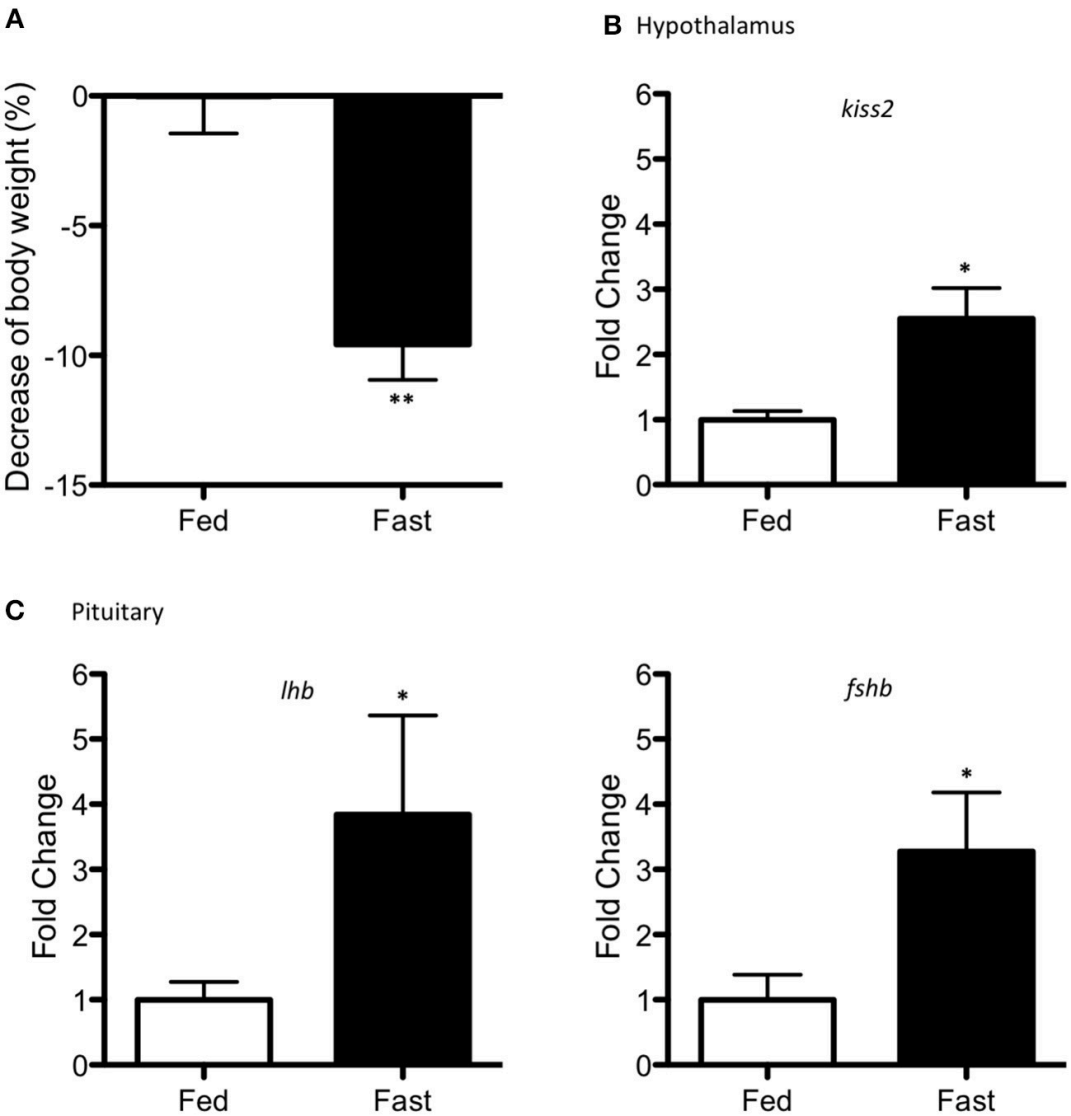
Effects of fasting (15 days period) in adult pejerrey. **(A)** Decrease of percentage in body weight during feeding and fasting conditions. **(B)** Expression levels of *kiss2* in the hypothalamus. **(C)** Expression levels of *lhb* and *fshb* in the pituitary. Results are represented as fold change after data normalization against β*-actin* levels Asterisks indicate statistically significant differences after the Student's *t*-test. **p < 0.01; *p < 0.05. Data shown are expressed as mean ± S.E.M. (*n* = 7–8).

Food deprivation resulted in a significant reduction of ~10 % in body weight (*p* < 0.001) in the starved adult pejerrey males (Figure 5A). Fasting increased mRNA levels of *kiss2* (*p* = 0.014) in the hypothalamus (Figure 5B) and increased levels of *lhb* and *fshb* in the pituitary (*p* = 0.032 and *p* = 0.048) (Figure 5C).

**Figure 6:**
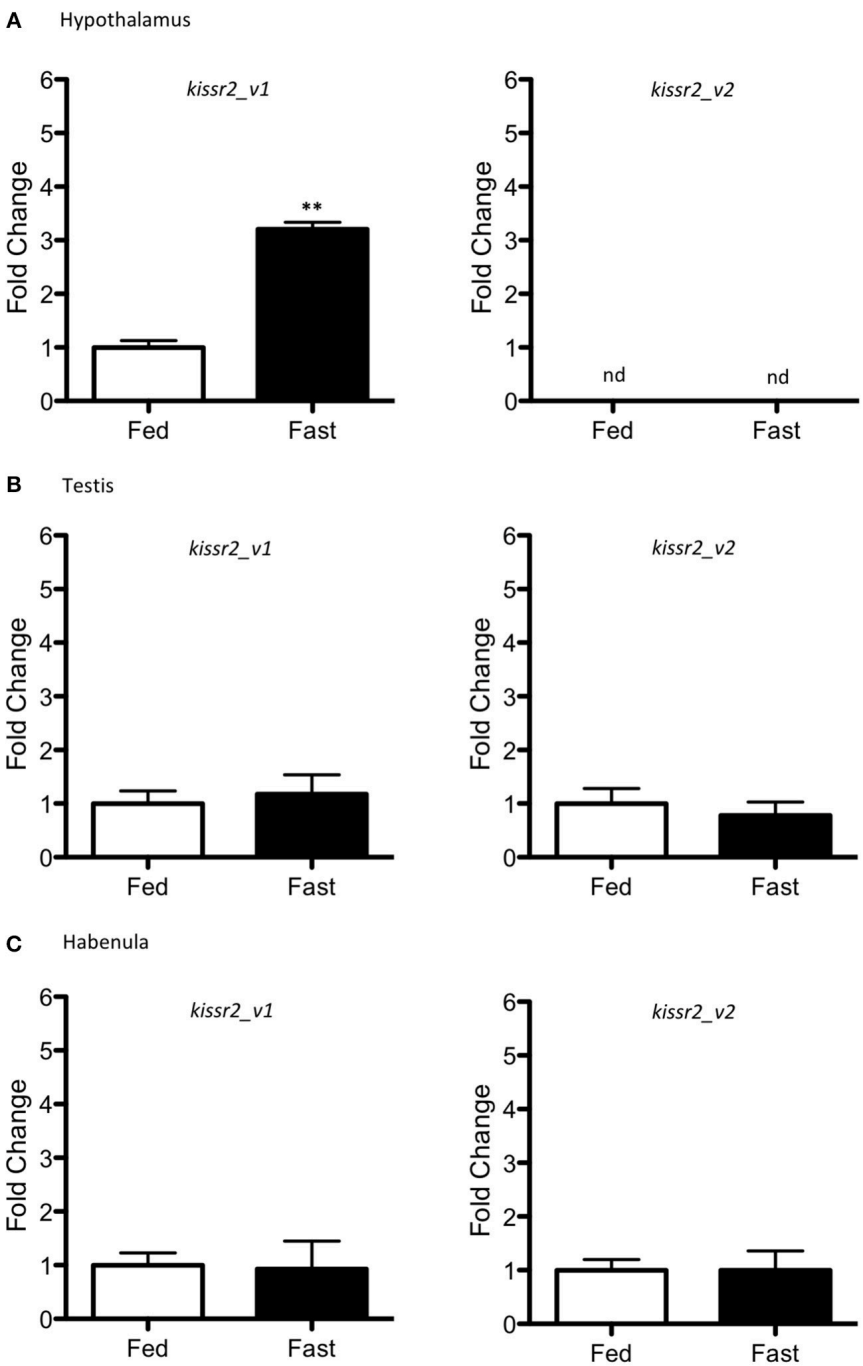
Gene expression in different tissues of male pejerrey. Expression levels of *kissr2_v1* and *kissr2_v2* isoforms in the hypothalamus **(A)**, testis **(B)**, and habenula **(C)**. Results are represented as fold change after data normalization against β*-actin* levels. Asterisks indicate statistically significant differences after the Student's *t*-test. **p < 0.01. n.d. non-detected. Data shown are expressed as mean ± S.E.M. (*n* = 7).

Changes in mRNA levels also were measured for *kissr2_v1* and *kissr2_v2* (Figure 6). In the hypothalamus, only *kissr2_v1* was detected in both control and fasted fish, with higher expression in fasted fish than controls (*p* = 0.001, Figure 6A). However, *kissr2_v2* was not detected in either case (Figure 6A). In testes and habenula both isoforms were observed in both feeding treatments, with no significant difference in expression level between isoforms (Figures 6B,C).

## Discussion

We have previously characterized two full-length cDNAs encoding *kissr2* and *kissr3* in pejerrey (29). In this study, we present additional analysis of their genomic structures and expression patterns in different tissues and under different experimental conditions. In agreement with a previous study of Senegalese Sole *kissr2* (20) and an *in silico* analysis in Nile Tilapia (35), the inferred structure of this gene in pejerrey consists of five exons and four introns. The pejerrey *kissr2* gene structure also is similar to its mammalian ortholog *KISSR1* (35). On the other hand, pejerrey *kissr3* presented six exons and five introns, similar to the situation observed in medaka (*Oryzias latipes*) and sea bass (36).

In pejerrey, *kissr2* and *kissr3* encode proteins of 379 and 369 aminoacids, respectively (29). These two paralogous proteins shared high similarity in their transmembrane domains, but low similarity in their ICL and ECL regions, similar to the situation observed in goldfish (37). Additionally, pejerrey Kissr2 and Kissr3 contain some typical features of the rhodopsin family, such as the NPXXY and DRY motifs (38).

Both receptors, Kissr2 and Kissr3 belong to the largest gene subfamily (Rhodopsin-like) within the GPCRs superfamily. Although exponential increase in our knowledge of crystallographic GPCRs structures during the last decade (39) helped to characterize many genes, lack of structural data still hampers a deep characterization of their function (40, 41). Indeed, solving protein structure remains a problematic issue due the limitations in protein production and purification, protein stability, and homogeneity (42) and, in some cases, low expression levels (43). Thus, computational tools are key to generate reliable protein structure modeling that may help in the characterization/elucidation of protein structure/function (31).

**Figure 7:**
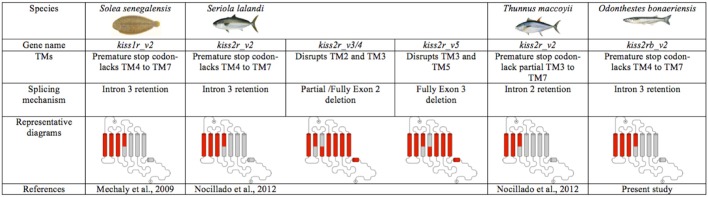
Different mechanisms of alternative splicing present in the *kissr2* gene of teleost fish due to intron retention and transmembrane domain (TM) deletion in *kissr2*.

It is also becoming increasingly evident that a high percentage of GPCRs undergo alternative splicing events (2); however, only in a few fish species alternative *Kissr* isoforms have been described (20–22). The present study documents alternative *Kissr2* isoforms in pejerrey (*Kissr2_v1* and *Kissr2_v2*). Furthermore, we show that the *kissr2_v2* isoform originates by retention of the entire intron 3, similar to the situation in the Senegalese sole (20). It is interesting to note that, in the case of foregut, the size of the smaller band looks shorter than the expected Kissr2_v1. At this moment, we cannot discard the possibility of the existence of a third isoform as it has been reported in other fish species (21, 22). Furthermore, in pejerrey, a species with two *kissr* paralogous genes, an alternative isoform of *kissr3* mRNA was also observed in male liver and hindgut, and the alternative isoform originates by retention of the entire intron 4. Unlike other species where alternative spliced variants originate by deletion of exons, such as yellowtail kingfish (21) and zebrafish (22), this mechanism is not present in pejerrey. However, more studies on the mechanism of splicing of the *kissr3* gene must be performed to get a better understanding of the role of splicing of this gene in different pejerrey tissues. The splicing mechanism, originating truncated isoforms in Kissr2 and/or Kissr3 in several teleost species appears to be a conserved feature and may represent a regulatory mechanism for controlling gene expression (44). We summarize the evidence reported so far for splicing events for these genes in teleost fish in Figure 7 and Supplementary Figure 5.

Alternative splicing is a mechanism that increases variability in protein products from a single gene (45). Although the physiological functions of alternative transcript isoforms are not completely understood, in some cases this mechanism could produce new products with defined functions (46). However, generation of variant products from alternative splicing not always results in functional proteins (47, 48). For example, if alternative splicing produces a large truncation, it is very unlikely that the synthesized polypeptide chain will fold properly (47). Moreover, most of the times, truncations are likely to produce dysfunctional proteins (49, 50). Accordingly, truncated GPCRs resulting from alternative splicing events are often retained in the endoplasmic reticulum (ER), preventing them to target the cell surface (24). For this reason, and to gain insights into the potential impact of alternative splicing on Kissr2 and Kissr3 structure/function, we generated structural models of the full-length receptors. Mapping of the missing regions of the spliced isoforms, Kissr2_v2 and Kissr3_v2 on the respective models, strongly suggests that the truncated receptors would be non-functional. Generally, in GPCRs, the missing structural elements (i.e., TM5–TM7) are involved either in ligand binding, signal transmission, or dimerization, thus they are critical for the correct functioning of the receptors (50). In some cases, mutations in rhodopsin receptors presented problems in the normal trafficking from the ER to the cell surface (51). Interestingly, in zebrafish the truncated isoform (called as KRBDP3) has been suggested to be functional from a regulatory point of view because it enters into the nucleus and presents ligand-independent transactivation activity (22). In the pejerrey case, however, we consider that the alternatively spliced receptor is non-functional, at least from the signaling point of view, similar to what has been observed in human KISSR1. Actually, single nucleotide mutations in this receptor were shown to be correlated with hypogonadotropic hypogonadism (HH) revealing that the alteration of the normal structure of this receptor will lead to lack of function (52, 53). Recently, a new alternative splicing mechanism in the 5′-untranslated regions (UTR) of *kissr2* gene in *Cynoglossus semilaevis* was described. In that study, genomic analysis of *kissr2* allowed the detection of three cDNA variants with common open reading frame (ORF) and 3′-UTR sequences, but with different 5′-UTR sequences (54). In this context, the alternative splicing events detected in pejerrey and in other teleost species (Figure 7) together with the existence of multiple promoters (54) seems to be a plausible mechanism to regulate *kissr2* expression in teleosts.

In mammals, the reproductive axis also is regulated by energy balance (35) and kisspeptin is related to food intake and growth (55, 56). Although several recent studies have proposed that kisspeptins regulate reproduction in teleosts, little is known about their role in the control of food intake and energy balance. The response of fasting in kisspeptin genes was first evidenced in the Senegalese sole in which an up-regulation of both *kiss2* and *kissr2* was observed during fasting (26). In the present study, not only *kiss2*, but also *kissr2_v1* was up-regulated after fasting while *kissr2_*v*2* showed no expression, suggesting that *kissr2_v1* is the functional form. Similar to that observed in the Senegalese sole (26) and a South American cichlid (57), an increase in pituitary Lh and Fsh mRNA was detected. It is important to say that we did not measure *kissr3* expression in fasting animals because not only *kissr3* gene was reported for *Senegalese sole* but also no *kissr3* expression was observed in pejerrey hypothalamus. Taken together, these findings suggest that a short period of food restriction can trigger the reproductive axis in fish. This is probably because in some fish species, the absence or limited food supply is associated with the beginning of a period where fish start their reproductive season. Our results also support previous observations of the possible orexigenic role of the *kissr2* (26, 27). However, in order to demonstrate the putative orexigenic role kisspeptins may an in fish, peptide-administration trials to test concomitant increase in foraging behavior need to be performed.

In summary, in this study, we have obtained the full genomic sequence of *kissr2* and *kissr3* in pejerrey and provided the first evidence for alternative splicing in both paralogous genes. Analysis of the Kissr2 and Kissr3 protein structures by 3D-models suggest that the alternative isoforms should give rise to non-functional GPCRs. The emergence of Next-generation sequencing (NGS), particularly RNAseq approaches, can offer promising avenues to discover novel isoforms of kisspeptin genes in other species and provide new information to study gene regulatory mechanism via alternative splicing in vertebrates.

## Author contributions

ASM and GMS conceived and designed the experiments. ASM, MT, AEM, MP, and AG performed the experiments. ASM, MT, AEM, ES, MP, AG, JV, and GMS analyzed the data. ASM and GMS wrote the paper. MT, AEM, AG, JV, and GO provided comments on the manuscript.

### Conflict of interest statement

The authors declare that the research was conducted in the absence of any commercial or financial relationships that could be construed as a potential conflict of interest.

## Supplementary material

Supplementary Table 1 Reaction efficiencies slope and correlation *r*^2^ values of real-time quantitative PCR assays. The efficiency and conditions for primer sets for kiss2, lhb, and, fshb were already reported in Tovar Bohórquez et al. (29) and Elisio et al. (58).

Supplementary Table 2 Predicted receptor-ligand interactions.

Supplementary Figure 1 **(A)** Schematic representation of a portion of *kissr2* gene in pejerrey showing the primer combinations used in this study. C1: kissr2-Ex3-4-F/kissr2-Ex4-R1, used to specifically amplify *kissr2_v1*, C2: kissr2-Int3-F/kissr2-Ex4-R, used to specifically amplify v2 isoform and C3: kissr2-Ex3-F/kissr2-Ex4-R, used to determine the presence of both isoforms in the tissue distribution experiments. **(B)** Representative agarose gel showing the expression of the *kissr2* gene in mature pejerrey. Messenger RNA of the two isoforms was detected by RT-PCR (C3 primer combination). Lane 1, 100 bp ladder (Ld); Lane 2, genomic DNA (gDNA); Lane 3, testis cDNA; Lane 4, gill cDNA; Lane 5, rostral brain cDNA; Lane 6, medial brain cDNA; Lane 7, testis RNA with DNase; Lane 8, gill RNA with DNase; Lane 9, rostral brain RNA with DNase; Lane 10, medial brain RNA with DNase. **(C)** A melt curve analysis was run after each amplification cycle to confirm specificity of the reaction of *kissr2_v1* (C1) and *kissr2_v2* (C2) and to confirm the presence of two peaks in the case of primers that are able to detect two isoforms (C3).

Supplementary Figure 2 Homology model of the Kissr2. Pejerrey Kiss1 sequence is shown in red.

Supplementary Figure 3 Homology model of the Kissr3. Pejerrey Kiss2 sequence is shown in red.

Supplementary Figure 4 Weblogo 3 (http://weblogo.threeplusone.com/) representation of the sequence alignments in teleost fish peptides. **(A)** Teleost Kiss1-15: pjKiss1-15: QDVSSYNLNSFGLRY. **(B)** Teleost Kiss2-12: pjKiss2-12: SKFNYNPFGLRF.

Supplementary Figure 5 Summary of alternative splicing due to intron retention and exon deletion detected in *kissr3* gene of teleost fish.
