# Supplementary material for: Evidence of Alternative Splicing as a Regulatory Mechanism for Kissr2 in Pejerrey Fish
Source: Front Endocrinol (Lausanne). 2018 Oct 17;9:604. doi: 10.3389/fendo.2018.00604 (PMC6200147; doi:10.3389/fendo.2018.00604)
Supplement: Supplementary Table 1 — Reaction efficiencies slope and correlation r2 values of real-time quantitative PCR assays. The efficiency and conditions for primer sets for kiss2, lhb, and, fshb were already reported in Tovar Bohórquez et al. (29) and Elisio et al. (58). [file Table_1.docx]

**Supplementary Table 1.** Reaction efficiencies slope and correlation r^2^ values of real-time quantitative PCR assays. The efficiency and conditions for primer sets for *kiss2*, *lhb* and, *fshb* were already reported in references 29 and 58.

| Transcript | Efficiency (%) | Slope | Correlation (r^2^) |
| --- | --- | --- | --- |
| *kissr2_v1* | 102 | -3.279 | 0.995 |
| *kissr2_v2* | 108 | -3.139 | 0.997 |
| *ef1* | 92 | -3.523 | 0.997 |
| *β-actin* | 95 | -3.442 | 0.999 |
