# Supplementary material for: Evidence of Alternative Splicing as a Regulatory Mechanism for Kissr2 in Pejerrey Fish
Source: Front Endocrinol (Lausanne). 2018 Oct 17;9:604. doi: 10.3389/fendo.2018.00604 (PMC6200147; doi:10.3389/fendo.2018.00604)
Supplement: Supplementary Table 2 — Predicted receptor-ligand interactions. [file Table_2.DOCX]

**Supplementary Table 2.** Predicted receptor-ligand interactions.

|  | H-bonds | Saline Bridge | Hydrophobic interactions |
| --- | --- | --- | --- |
| Kiss1-Kissr2 | Q1-E35, S4/5-Q38, N7-R195, S10-Y197, Y15-Q125/N311 | R14-E208 | V3-P194, Y6-Y192, F11-Y304/Y106, Y15/Y315 |
| Kiss1-Kissr3 | R11-Y185 |  | F3-Y178, F5-W35/Y289, P7-L283, F8-Y95, L10-L283, F12-I193 |
| Kiss2-Kissr2 | L2-Y297, N4-R195, Q9-Q291, R11-Y192 |  | F3-Y192, F5-F41/Y304, F12-Y166 |
| Kiss2-Kissr3 | S10/R14-Q183 | D2-R284, R14-E188 | Y6-Y178, F11-Y95/Y289, L13-Y300 |
