# Supplementary figures and images for: Evidence of Alternative Splicing as a Regulatory Mechanism for Kissr2 in Pejerrey Fish
Source: Front Endocrinol (Lausanne). 2018 Oct 17;9:604. doi: 10.3389/fendo.2018.00604 (PMC6200147; doi:10.3389/fendo.2018.00604)

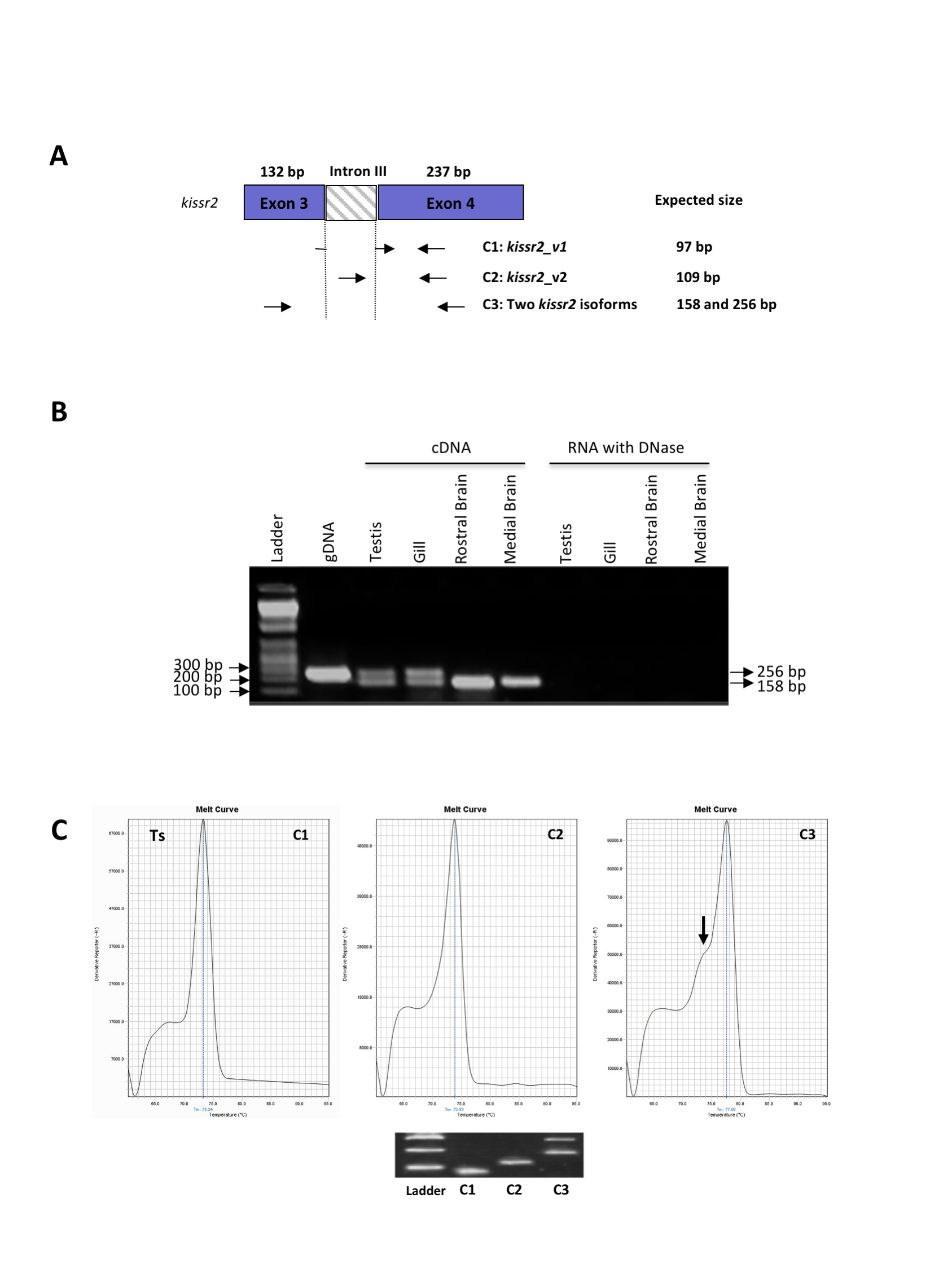

Supplement: Supplementary Figure 1 — (A) Schematic representation of a portion of kissr2 gene in pejerrey showing the primer combinations used in this study. C1: kissr2-Ex3-4-F/kissr2-Ex4-R1, used to specifically amplify kissr2_v1, C2: kissr2-Int3-F/kissr2-Ex4-R, used to specifically amplify v2 isoform and C3: kissr2-Ex3-F/kissr2-Ex4-R, used to determine the presence of both isoforms in the tissue distribution experiments. (B) Representative agarose gel showing the expression of the kissr2 gene in mature pejerrey. Messenger RNA of the two isoforms was detected by RT-PCR (C3 primer combination). Lane 1, 100 bp ladder (Ld); Lane 2, genomic DNA (gDNA); Lane 3, testis cDNA; Lane 4, gill cDNA; Lane 5, rostral brain cDNA; Lane 6, medial brain cDNA; Lane 7, testis RNA with DNase; Lane 8, gill RNA with DNase; Lane 9, rostral brain RNA with DNase; Lane 10, medial brain RNA with DNase. (C) A melt curve analysis was run after each amplification cycle to confirm specificity of the reaction of kissr2_v1 (C1) and kissr2_v2 (C2) and to confirm the presence of two peaks in the case of primers that are able to detect two isoforms (C3). [file Image_1.TIF]

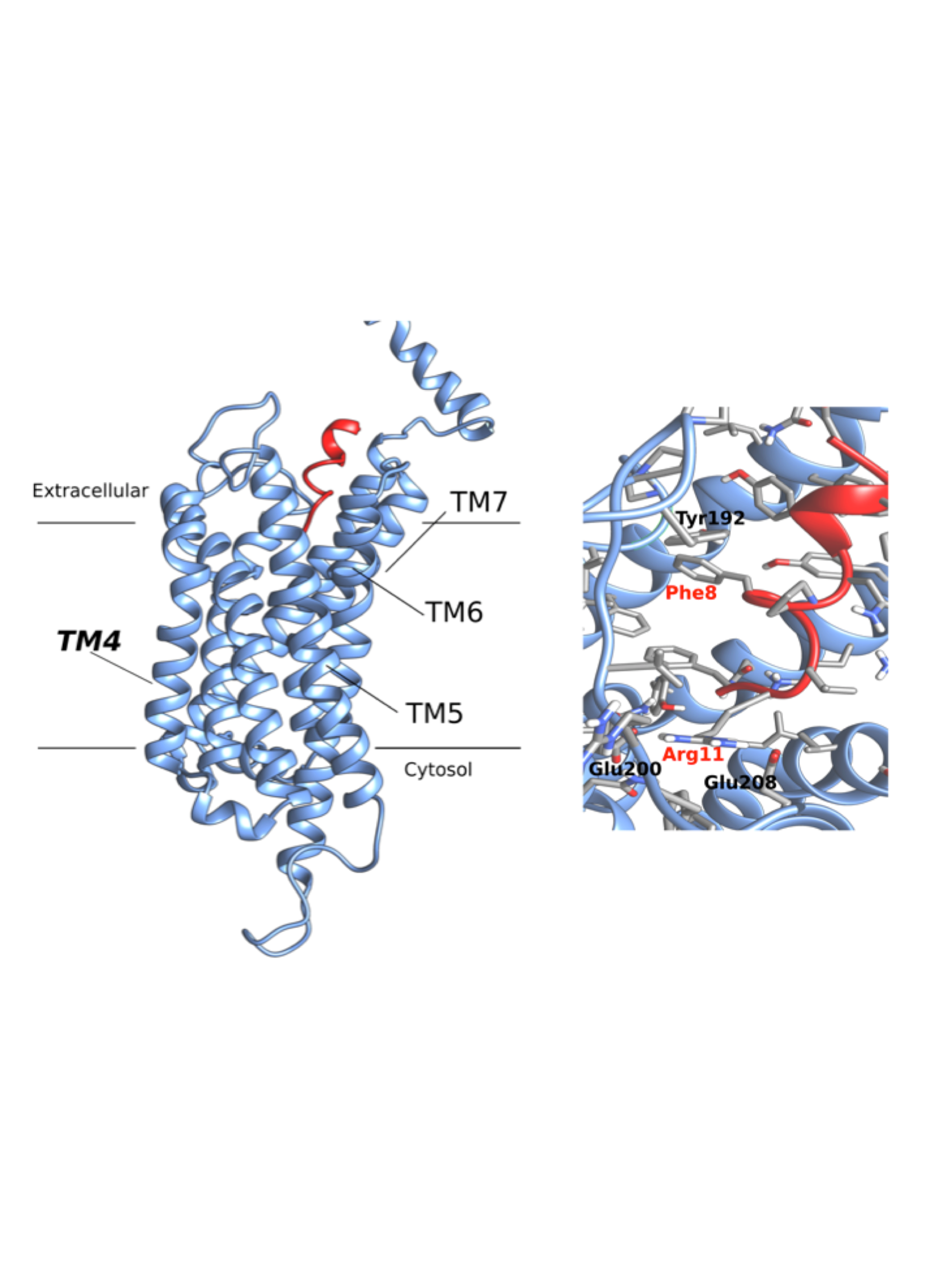

Supplement: Supplementary Figure 2 — Homology model of the Kissr2. Pejerrey Kiss1 sequence is shown in red. [file Image_2.TIFF]

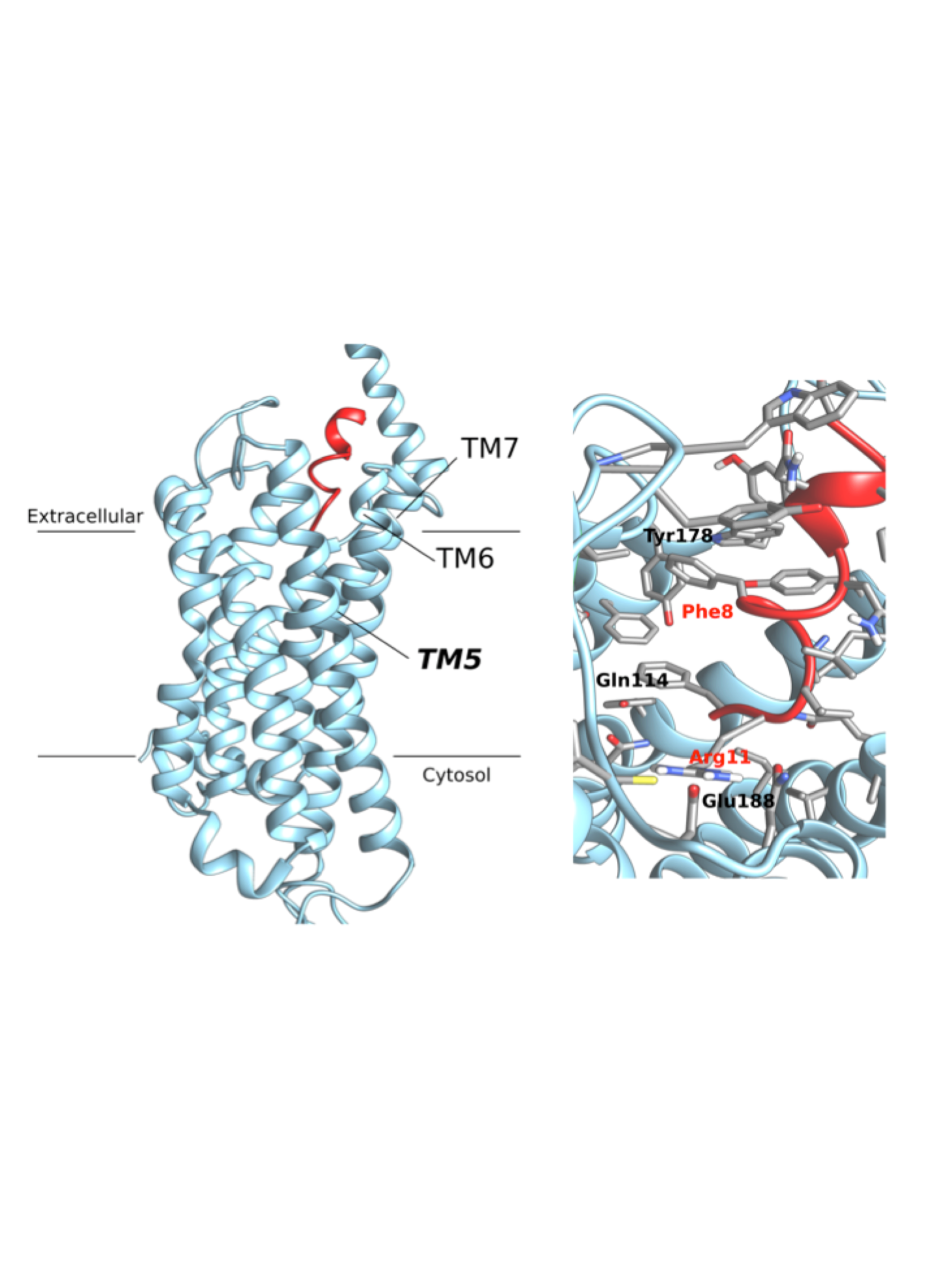

Supplement: Supplementary Figure 3 — Homology model of the Kissr3. Pejerrey Kiss2 sequence is shown in red. [file Image_3.TIFF]

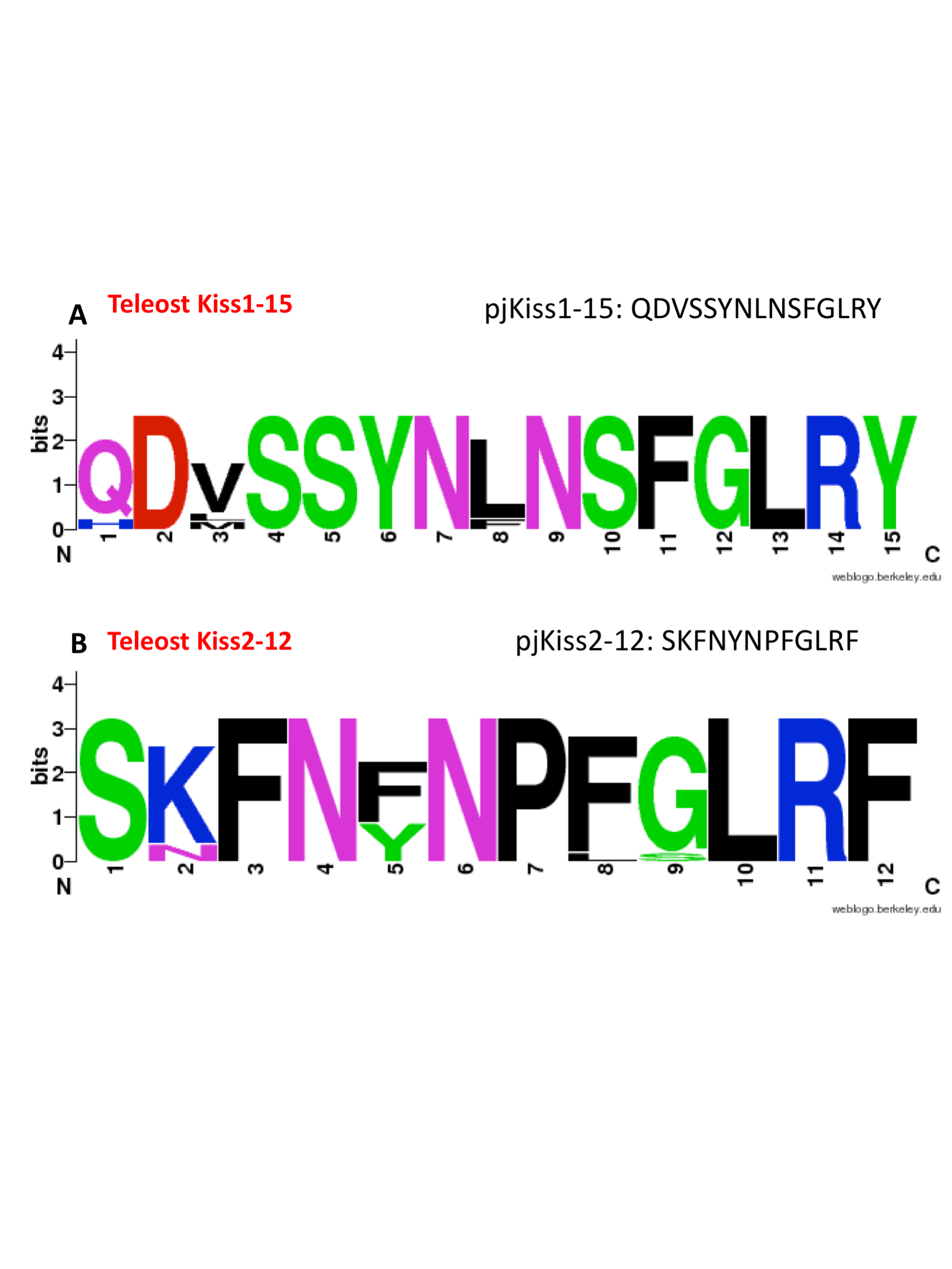

Supplement: Supplementary Figure 4 — Weblogo 3 (http://weblogo.threeplusone.com/) representation of the sequence alignments in teleost fish peptides. (A) Teleost Kiss1-15: pjKiss1-15: QDVSSYNLNSFGLRY. (B) Teleost Kiss2-12: pjKiss2-12: SKFNYNPFGLRF. [file Image_4.TIFF]

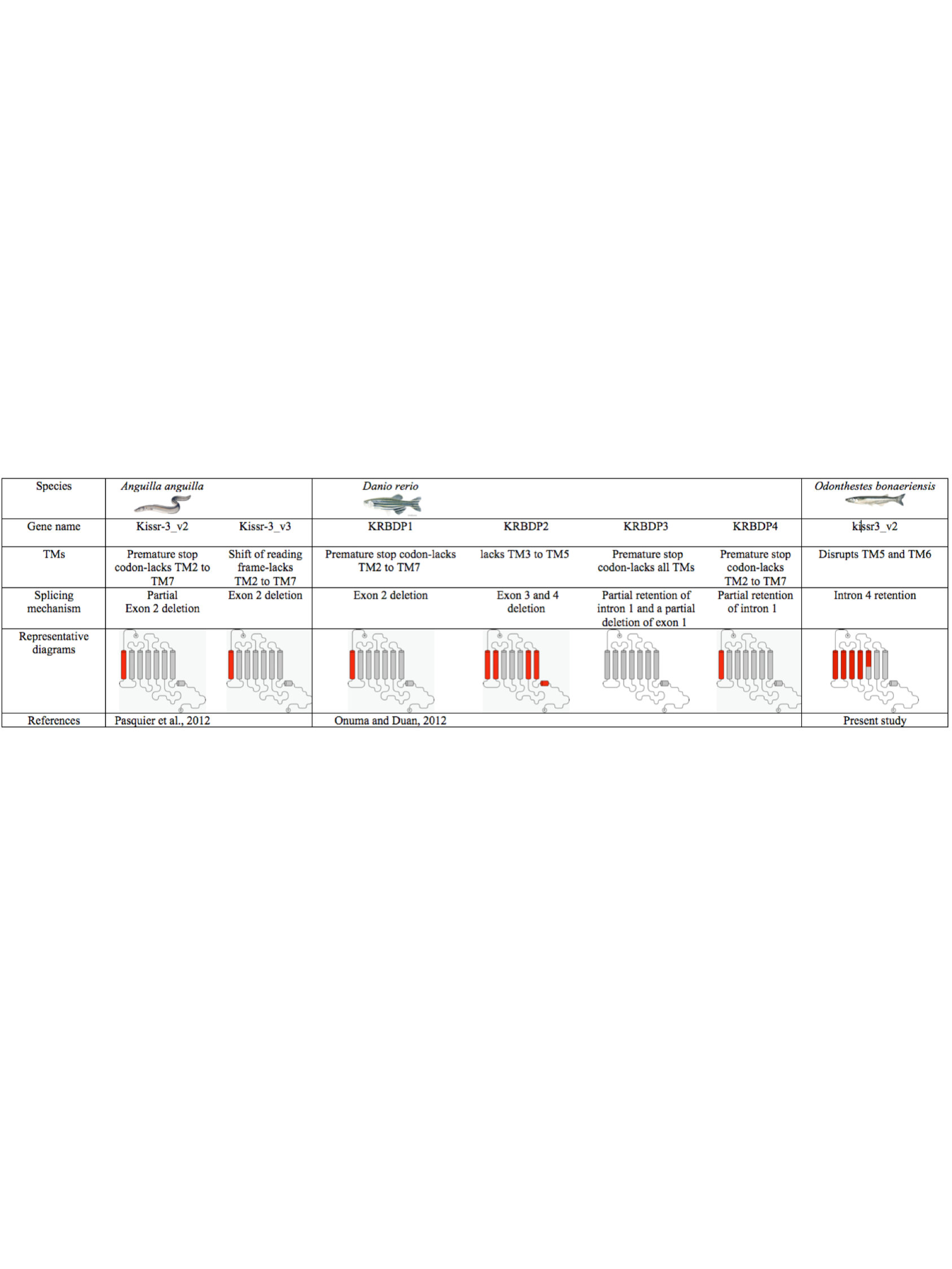

Supplement: Supplementary Figure 5 — Summary of alternative splicing due to intron retention and exon deletion detected in kissr3 gene of teleost fish. [file Image_5.TIFF]
